# A Truncated NRIP1 Mutant Amplifies Microsatellite Instability of Colorectal Cancer by Regulating MSH2/MSH6 Expression, and Is a Prognostic Marker of Stage III Tumors

**DOI:** 10.3390/cancers13174449

**Published:** 2021-09-03

**Authors:** Pascale Palassin, Marion Lapierre, Samuel Pyrdziak, Antoine Wagner, Régine Stehle, Carole Corsini, Jacqueline Duffour, Sandrine Bonnet, Abdelhay Boulahtouf, Carmen Rodriguez, Alexandre Ho-Pun-Cheung, Evelyne Lopez-Crapez, Florence Boissière-Michot, Frédéric Bibeau, Simon Thezenas, Nabila Elarouci, Janick Selves, Jean-Sébastien Hoffmann, Paul Roepman, Thibault Mazard, Olivier Buhard, Alex Duval, Stéphan Jalaguier, Vincent Cavaillès, Audrey Castet-Nicolas

**Affiliations:** 1 IRCM, Institut de Recherche en Cancérologie de Montpellier, INSERM, U1194, Institut Régional du Cancer de Montpellier, Université de Montpellier, F-34298 Montpellier, France; p-palassin@chu-montpellier.fr (P.P.); marion.lapierre@inserm.fr (M.L.); samuel.pyrdziak@evotec.com (S.P.); antoine.wagner@orange.fr (A.W.); regine.stehle@gmail.com (R.S.); c-corsini@chu-montpellier.fr (C.C.); jacqueline.duffour@sfr.fr (J.D.); sandrine.bonnet@inserm.fr (S.B.); abdel.boulahtouf@inserm.fr (A.B.); carmen.rodriguez@icm.unicancer.fr (C.R.); evelyne.crapez@icm.unicancer.fr (E.L.-C.); bibeau-f@chu-caen.fr (F.B.); simon.thezenas@icm.unicancer.fr (S.T.); thibault.mazard@icm.unicancer.fr (T.M.); stephan.jalaguier@inserm.fr (S.J.); audrey-castet@chu-montpellier.fr (A.C.-N.); 2 Translational Research Unit, Institut du Cancer de Montpellier, F-34298 Montpellier, France; alexandre.ho-pun-cheung@icm.unicancer.fr (A.H.-P.-C.); florence.boissiere@icm.unicancer.fr (F.B.-M.); 3 Programme Cartes d’Identité des Tumeurs (CIT), Ligue Nationale Contre Le Cancer, F-75013 Paris, France; nabila.elarouci@icm-institute.org; 4 Département de Pathologie, IUCT, Institut Universitaire du Cancer de Toulouse—Oncopôle, 31059 Toulouse, France; selves.j@chu-toulouse.fr (J.S.); jean-sebastien.hoffmann@inserm.fr (J.-S.H.); 5 Agendia NV, 1043NT Amsterdam, The Netherlands; p.roepman@hartwigmedicalfoundation.nl; 6 Centre de Recherche Saint-Antoine, Equipe Instabilité des Microsatellites et Cancer, Unité Mixte de Recherche Scientifique 938 et SIRIC CURAMUS, INSERM, Sorbonne Université, 75012 Paris, France; olivier.buhard@inserm.fr (O.B.); alex.duval@inserm.fr (A.D.); 7 Centre Hospitalo-Universitaire Montpellier, Département de Pharmacie Clinique, 34295 Montpellier, France; 8 Unité de Formation et de Recherche des Sciences Pharmaceutiques et Biologiques, 34090 Montpellier, France

**Keywords:** colorectal cancer, mismatch repair, microsatellite instability, RIP140/NRIP1, patient prognosis

## Abstract

**Simple Summary:**

The alteration of mismatch repair (MMR) genes leads to microsatellite instability and plays a key role in colorectal cancer (CRC) pathogenesis and prognosis. The transcription factor NRIP1 is involved in intestinal tumorigenesis and is a good prognostic marker in CRC. In this study, we demonstrate that NRIP1 induces MSH2 and MSH6 MMR gene transcription and reduces microsatellite instability. A dominant-negative truncated NRIP1 mutant amplifies the MMR-deficient phenotype and appears as a key player in MSI-driven tumorigenesis since it significantly correlates with a short overall survival of patients with advanced CRC, especially MLH1-deficient ones.

**Abstract:**

Microsatellite instability (MSI) is related to the alteration of mismatch repair (MMR) genes and plays a key role in colorectal cancer (CRC) pathogenesis. We previously reported that the transcription factor Nuclear Receptor Interacting Protein 1 (NRIP1) is involved in sporadic intestinal tumorigenesis. The aim of this study was to decipher its role in MSI CRC. By using different mouse models and engineered cell lines, we demonstrated that NRIP1 increased MSH2 and MSH6 MMR gene transcription and mRNA/protein levels. In human CRC cells, NRIP1 expression was associated with decreased MSI and the hypermutator phenotype, and with resistance to chemotherapy drugs. Using a cohort of 194 CRC patients, we detected in 22% of the cases a MSI-induced frameshift mutation in the NRIP1 coding sequence. This genetic alteration generates a truncated protein with a dominant negative activity that increased human CRC cell proliferation and impaired the regulation of MSH2 and MSH6 gene expression. Moreover, the NRIP1 mutant correlated with a decreased overall survival of patients with advanced CRC, especially when MLH1-deficient. By decreasing the expression of MSH2 and MSH6 gene expression, the NRIP1 variant may amplify MLH1-dependent CRC progression and behave as a new prognostic marker of advanced MSI CRC.

## 1. Introduction

Colorectal cancer (CRC) is one of the most common cancers worldwide [1]. Genetic instability, including chromosomal and microsatellite instability (MSI), is a key event in CRC pathogenesis. Indeed, MSI is observed in 15% of CRC, due to loss of expression of at least one of the genes involved in the mismatch repair (MMR) system implicated in error correction during DNA replication. Within the MMR system, the DNA recognition complex MutSα consists of MSH2-MSH6 heterodimers. Following mismatch detection, the MutSα complex binds to the execution complex MutLα (MLH1 and PMS2) to signal the need to carry out excision and re-synthesis of the mismatched DNA [2]. MMR impairment leads to hypermutated tumors that accumulate frameshift mutations in the genome [3]. In sporadic MSI CRC, *MLH1* gene promoter hypermethylation is the main cause of MMR deficiency (dMMR) [4]. Moreover, an inherited component is observed in about 25% of CRCs, and this includes Lynch syndrome (LS), which is due to an autosomal dominant heterozygous constitutional mutation in one of the MMR genes [5].

In comparison with microsatellite stable (MSS) tumors, stage I/II CRC with high-level MSI (MSI-H) appears less aggressive and exhibits a favorable prognosis [6]. This seems to be linked to local inflammation [7] in response to the production of highly immunogenic frameshift peptides [8]. Adjuvant chemotherapy with 5-fluorouracil (5-FU) is inefficient in patients with stage II dMMR CRC [9]. Conversely, chemotherapy regimens that combine folinic acid, 5-FU and oxaliplatin are effective in stage III or metastatic MSI-H cancers, similar to their MSS countertype, although those patients may have worse [6] or better [10] outcomes. Interestingly, recent studies suggested high efficacy of immune checkpoint inhibitors in chemoresistant dMMR/MSI metastatic CRC, related to their high tumor mutational burden [11]. However, few genetic markers have demonstrated prognostic or predictive value for the treatment decision in MSI CRC, such as the *V600E* mutation of B-type raf proto-oncogene (BRAF) [12] and the HSP110 T17 deletion [13], leaving room to find new candidates.

The transcription coregulator Nuclear Receptor-Interacting Protein 1 (NRIP1), also known as Receptor Interacting Protein of 140 kDa (RIP140), is a major regulator of various nuclear signaling pathways such as nuclear hormone receptors (NR) or other transcription factors, as E2F [14,15,16]. NRIP1 mainly acts as a transcriptional repressor by means of four inhibitory domains, recruiting histone deacetylases or CtBPs (C-terminal Binding Proteins) [17], and several post-translational modifications play important roles in controlling its subcellular location and repressive activity [18]. NRIP1 also positively regulates the transcriptional activity of NRs, including ERR (Estrogen Receptor Related Receptor), via Sp1 sites [19]. Invalidation of the *Nrip1* gene (RIPKO mice) has revealed a role in ovulation, metabolism, cognition and inflammation [20]. Moreover, a single nucleotide polymorphism, which introduces an amino-acid change in the *NRIP1* coding sequence, was associated to various pathologies, including endometriosis [21] and lung cancer [22]. We previously reported that NRIP1 negatively regulates the Wnt/β-catenin signaling pathway and exerts an important role in normal and malignant development of intestinal epithelium [23]. In CRC, NRIP1 is a good prognostic marker because its expression is significantly correlated with better overall survival (OS) [23,24]. These data dealing with the role of NRIP1 in CRC have been obtained mainly in a context of sporadic CRC, independently of the MSI status. Since MMR deficiency plays a key role in intestinal tumorigenesis as above detailed, we wanted to explore the role of NRIP1 in this subgroup of CRC and investigate its implication in the control of MMR gene expression by combining the use of human CRC cell lines and genetically modified mouse models.

We found that NRIP1 increases *MSH2* and *MSH6* gene expression, alters microsatellite stability and mutation repair efficiency and modifies their response to cytotoxic drugs. Moreover, in MSI CRC cells and tumors, a frameshift mutation in the NRIP1 coding sequence impairs its biological activity. This mutation is associated with poor OS of patients with stage III CRC, especially those with MLH1 deficiency (dMLH1). We propose that through the modulation of MutSα levels, NRIP1 actively participates in the dMMR phenotype of intestinal epithelial cancer cells, and represents a promising prognostic marker to adapt the management of patients with MSI CRC.

## 2. Materials and Methods

### 2.1. Mouse Models

C57BL/6J *Nrip1*^−/−^ (RIPKO) mice were provided by M.G. Parker [25]. C57BL/6/129 NRIP1 transgenic (RIPTg) mice were previously obtained using Speedy Mouse Technology (Nucleis) by insertion of a copy of human Nrip1 cDNA at the HPRT locus as described [23]. The selection of the ES cell clones with homologous recombination was realized with hypoxanthine-aminopterin-thymidine-supplemented (HAT-supplemented) medium. Targeted ES cells were injected into C57BL/6-derived blastocysts before recipient female transplantation and backcrossing. Genotyping of animals was realized by qPCR.

### 2.2. Plasmids

The pRL-CMV-renilla and pGL vectors were from Promega (Charbonnieres, France). pEF-cmyc-RIP140 and pEGFP-RIP140 were previously described [26,27]. pEF-cmyc-RIP^MSI^ was generated using QuikChange (Agilent Technologies, Santa Clara, CA, USA). pEF-cmyc-RIP^MSI^ was digested with AflII and EcoRV enzymes, and the resulting insert was cloned into pEGFP-RIP140 to create pEGFP-RIP^MSI^. Green Fluorescent Protein (GFP), GFP-RIP140 and GFP-RIP140^MSI^ were PCR-amplified and cloned in the pTRIPZ vector previously digested with AgeI and MluI to create pTRIPZ-GFP, pTRIPZ-RIP140 and pTRIPZ-RIP^MSI^, respectively. All the engineered PCR constructs were sequenced.

### 2.3. Cell Culture and Transfections

Mouse embryonic fibroblasts (MEFs) derived from wild-type (WT) and RIPKO mice were grown in DMEM-F12 medium supplemented with 10% FCS, 100 U/mL penicillin, 100 mg/mL streptomycin and 100 mg/mL sodium pyruvate, transfected using Lipofectamin 2000 (ThermoFisher Scientific, Waltham, MA USA) with the pTRIPZ plasmids encoding GFP alone, GFP-RIP140 or GFP-RIP140^MSI^ and selected with 40 µg/mL puromycin. Different human CRC cell lines including SW480, HT29, RKO and LoVo cells were used and cultured as recommended by the ATCC. Some of these cell lines were used because they exhibited high levels of endogenous NRIP1. Stably transfected HCT116-GFP and HCT116-RIP140 were grown in McCoy medium completed as the MEFs but selected by 750 µg/mL G418 [23]. Two HCT116 cell lines with different levels of *NRIP1* gene expression (low in HCT116LR and high in HCT116HR) were used in some experiments. Transfections with siRNAs (Supplementary Table S1) were performed using INTERFERin on cells seeded the day before (3 × 10^5^ cells in 6-well plates) and validated by RT-qPCR.

### 2.4. Luciferase and ChIP Assays

The MSH2 and MSH6 luciferase reporter and NRIP1 expression vectors were previously described [26,27,28,29]. HCT116 cells were transfected in 96-well plates (2.5 × 10^4^ cells per well) 24 h prior to DNA transfection with Jet-PEI (275 ng of total DNA). Increasing doses of pEF-c-myc-RIP140 or pEF-c-myc-RIP^MSI^ were cotransfected with the pGL3-MSH2-Luc or the Sp1 mutant pGL3-MSH2m1-Luc (kind gifts of E. Huang [28]). Similar experiments were performed with the pGL3-MSH6-Luc reporter vector and a Sp1 mutant pGL3-MSH6M1-2/7-Luc (kind gifts of R.D. Kolodner [29]). The pRL-CMV-renilla plasmid (Promega, Charbonnières-les-Bains, France) was used to normalize transfection efficiency. Firefly luciferase values were measured and normalized by the Renilla luciferase activity. Values were expressed as the mean ratio of luciferase activities.

ChIP assays for proteins at the *MSH2* and *MSH6* promoters were performed in HT29 cells using the CHIP-IT kit (Active Motif, Carlsbad, CA, USA). Sonicated chromatin was immunoprecipitated with antibodies against IgG (sc-3739, Santa Cruz Biotechnology, Inc, Heidelberg, Germany), H3pan (CC16310135, Diagenode, Liège, Belgium) and NRIP1 (ab42126, Abcam, Paris, France). Immunoprecipitated DNA was amplified by qPCR using the primers listed in Supplementary Table S1.

### 2.5. Cell Proliferation and Cytotoxicity Assays

Cells were seeded in quadruplicate in 96-well plates at a density of 2 × 10^3^ cells per well. At the indicated time, 0.5 mg/mL of 3-(4,5-dimethylthiazol-2-yl)-2,5-diphenyltetrazolium bromide (MTT) (Sigma-Aldrich, Saint-Quentin, France) were added and incubated at 37 °C for 4 h. Formazan crystals were solubilized in DMSO and absorbance read at 560 nm on a spectrophotometer. Results were normalized to the cell density at day 1. For cytotoxicity assays, cells were seeded in quadruplicate in a 96-well plate (2.5 × 10^3^ cells per well) and exposed the day after to increasing concentrations of cytotoxic drugs including 5-fluorouracil, SN38, oxaliplatin (Sigma-Aldrich, Saint-Quentin, France) or to vehicle alone. The cells were exposed to the drug during six days, and cell viability was quantified each day using MTT assay. Values were normalized to the mean optical density of the control for each day.

### 2.6. Reverse Transcription-Real-Time Quantitative PCR (RT-qPCR)

Total RNA was extracted with the Quick-RNA kit (Zymo Research, Irvine, CA, USA), and 1 µg was subjected to reverse-transcription using qScript cDNA SuperMix (QuantaBio, VWR, Strasbourg, France). RT-qPCR were performed with the Roche LightCycler 480 instrument and the PerfeCTa SYBR Green FastMix (QuantaBio, VWR, Strasbourg, France) and were carried out in a final volume of 10μL using 0.25 µL of each primer (25 μM), 5 μL of the supplied enzyme mix, 2.5 μL of H_2_O and 2 μL of the template diluted at 1:10 (See Supplementary Table S1 for primer sequences). After pre-incubation at 95 °C, runs corresponded to 35 cycles of 15 s each at 95 °C, 5 s at 60 °C and 15 s at 72 °C. Melting curves of the PCR products were evaluated using the LightCycler 480 SW1.5 software to eliminate amplification of unspecific products [23]. Results were normalized to the mouse *RS9* or human *28S* housekeeping gene transcripts.

### 2.7. MSI and HPRT Mutation Assay

MMR status was determined by fluorescent multiplex PCR-based analysis of five microsatellite markers [30]. For HPRT mutation assay, HCT116-GFP and HCT116-RIP140 cells were incubated in HAT medium (Life Technologies, Paisley, UK) to eliminate cells harboring preexisting hypoxanthine-guanine phosphoribosyl transferase (HPRT) mutants. After 3 days, cells were seeded in Petri dishes, incubated with 20 µM O^6^-benzylguanine (Sigma-Aldrich, Saint-Quentin, France) for 2 h to deplete O-6-methylguanine-DNA methyltransferase (MGMT) and then supplemented or not with 1 µM methyl-nitro-nitroso-guanidine (MNNG) (TCI Europe) for 1 h. Cells were seeded in 6-well plates (200 cells/well), and cloning efficiency was analyzed 10 days later. In parallel, 10^5^, 2 × 10^5^ or 5 × 10^5^ cells per well were plated with 5 µg/mL 6-thioguanine (6-TG, Sigma-Aldrich, Saint-Quentin, France) and cultured for 30 days. After staining with crystal violet, the mutation frequency was calculated (number of colonies/cloning efficiency × 10^5^).

### 2.8. Immunoblotting

Whole cell extracts prepared in RIPA buffer (40 µg) were analyzed by western blotting using antibodies against NRIP1 (ab42125), MSH2 (ab70270) and MSH6 (ab92471) (Abcam, Paris, France). Signals were revealed using a rabbit peroxidase-conjugated secondary antibody (1/5000, A6154 Sigma-Aldrich, Saint-Quentin, France) and enhanced chemiluminescence (ECL-RevelBlotPlus; GE Healthcare) according to the manufacturer’s instructions. Normalization was conducted relative to β-actin (A3854; Sigma-Aldrich, Saint-Quentin, France).

### 2.9. Immunofluorescence (IF) and Immunohistochemistry (IHC) Analysis

For IF, cells were fixed with 3.7% paraformaldehyde, permeabilized and incubated with primary antibodies against NRIP1 (1:100, ab42126), MSH2 (1:1000, ab70270) and MSH6 (1:1000 ab92471) (Abcam, Paris, France) at 4 °C overnight, diluted in PBS-Tween, 3% BSA and then with Alexa-conjugated secondary rabbit IgG (AF488, AF594, AF546, 1/400, Invitrogen) at room temperature for 1 h. Cells were counterstained with Hoechst (1/1000, Sigma-Aldrich, Saint-Quentin, France), and slides were mounted with Mowiol (Sigma-Aldrich, Saint-Quentin, France). Staining quantification was performed at x40 magnification using the AxioVision ZEN 2 software (Carl Zeiss).

For IHC, following incubation in citrate buffer solution, paraffin-embedded tissue sections were incubated with anti-MSH2 (ab70270) and -MSH6 (ab92471) (Abcam, Paris, France) antibodies at 4 °C overnight, and then with a peroxidase-conjugated secondary antibody (Jackson Immunoresearch) at room temperature for 1 h, followed by 3,3’-Diaminobenzidine revelation (DAKO cytomation). Sections were counterstained with hematoxylin and images taken using NanoZoomer NDP.view2 software (Hamamatsu Photonics).

For IHC of human CRC samples, two TMA were obtained from the Biological Resources Center (BB-033-00059) of the Institute for Cancer of Montpellier (ICM). They were incubated with antibodies against NRIP1 (ab42126, Abcam, Paris, France), MSH2 (clone FE11, Agilent Dako, Santa Clara, CA, USA) and MSH6 (ab92471 Abcam, Paris, France) using the Autostainer Link48 platform (Agilent Dako, Santa Clara, CA, USA). Signal amplification was conducted with the Flex+ system. The H-score was determined for each spot, by taking into account the percentage of positive tumor cells and the staining intensity [31].

### 2.10. Patients and Specimens

MSI CRC samples at different stages were obtained from ICM (*n* = 80 patients), Saint Antoine Hospital (Paris) (*n* = 68 patients) and the Cancer Research Center of Toulouse (CRCT) (*n* = 46 patients), in accordance with the French regulatory laws. All surgical CRC specimens were dMMR (presence of MSI and/or loss of MMR protein expression in the tumor). For samples from ICM and CRCT, the frameshift mutation in the NRIP1 coding sequence (RIP^MSI^) was detected by Illumina HiSeq4000 deep-sequencing (Integragen genomics, France) using specific primers (Supplementary Table S1) and DNA extracted using the QIAamp DNA FFPE kit (Qiagen, Hilden, Germany). For the Saint Antoine hospital cohort, RNA-seq analysis was performed as described [32]. *NRIP1*, *MSH2* and *MSH6* mRNA expression was analyzed using published DNA microarray data from different cohorts of patients with CRC [3,33,34].

### 2.11. Statistical Analysis

All experiments were realized independently at least three times. Results were presented as the mean ± standard deviation (SD). For continuous parameters, descriptive analyses were performed using median and ranges and compared with the Kruskal–Wallis or Wilcoxon test. Frequencies and percentages were used for categorical variables that were compared using the Chi2 or Fisher’s exact tests. The association of clinical and mutation/tumor parameters with OS was assessed using univariate Cox proportional hazard regression analyses (“survival” R package). Censoring was systematically applied at 5 years, and survival was described using Kaplan–Meier curves. Other statistical analyses were performed using the Mann–Whitney, Spearman or log-rank test and STATEL, STATA 13.1 or GraphPad Prism version 8. Differences between groups were considered statistically significant at *p* < 0.05.

## 3. Results

### 3.1. NRIP1 Regulates MSH2 and MSH6 Gene Expression in Mouse Models

**Figure 1:**
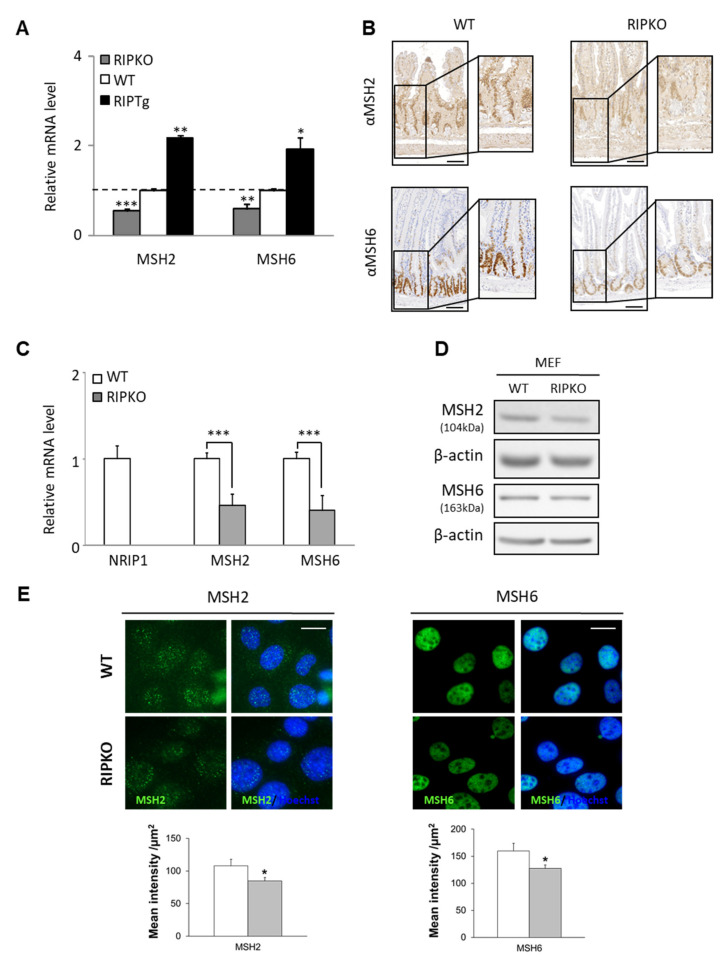
NRIP1 regulates *MutSα* gene expression in mouse tissue and cells. (**A**) RT-qPCR analysis of *Msh2* and *Msh6* expression in whole small intestine isolated from RIPKO, wild-type (WT) and RIPTg mice. Results represent the fold change ± SD compared with WT mice (dotted line) after normalization to *Rs9* level; * *p* < 0.05, ** *p* < 0.01, *** *p* < 0.001 (Student *t*-test). (**B**) MSH2 and MSH6 expression (IHC) in intestinal epithelium of RIPKO mice and WT littermates (x10). Scale bar, 100 µm. (**C**) *Msh2* and *Msh6* expression in immortalized WT and RIPKO MEFs; *** *p* < 0.001 (Mann–Whitney test). (**D**) Western blot analysis of MSH2 and MSH6 protein expression in WT and RIPKO MEFs (left), and data quantification (right; in arbitrary units, AU). (**E**) MSH2 and MSH6 immunofluorescence analysis in WT and RIPKO MEFs (40×), and quantification (lower panels) shown as the mean intensity per µm^2^ ± SD; Scale bar, 20 µm; * *p* < 0.05 (Student *t*-test).

To determine the NRIP1 role in *MMR* gene expression, we analyzed transgenic mice in which *Nrip1* was knocked-out (RIPKO mice) or overexpressed (RIPTg mice). We previously used these mice models and validated by immunofluorescence that the NRIP1 protein was undetectable in the RIPKO mice and overexpressed in the nuclei of all intestinal epithelial cells in the RIPTg mice as compared with wild-type (WT) animals [23]. Compared with WT littermates, *Msh2* and *Msh6* mRNA levels were lower in the whole small intestine of RIPKO mice, whereas they were increased in RIPTg mice (Figure 1A). These effects appeared to be specific because the expression of other MMR genes, such as *Mlh1*, was not affected by *Nrip1* deletion and overexpression (data not shown). The decreased *Msh2* and *Msh6* gene expression in the intestinal epithelium of RIPKO mice was confirmed by IHC (Figure 1B).

Msh2/Msh6 gene and protein (by western blotting and immunofluorescence) levels were also significantly reduced in MEFs obtained from RIPKO mice, compared with WT MEFs (Figure 1C–E). These results showed that in mouse intestinal epithelium, NRIP1 increases the expression of the two MutSα components: Msh2 and Msh6.

### 3.2. NRIP1 Regulates MSH2/MSH6 Gene Transcription in Human CRC Cells

**Figure 2:**
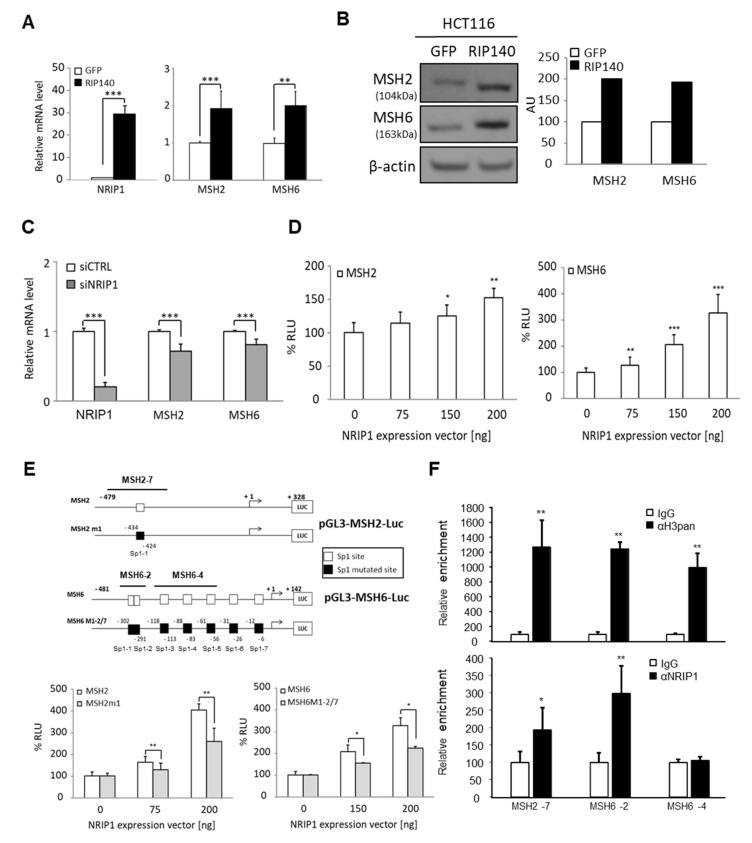
NRIP1 regulates *MUTSα* gene expression in human CRC cells. (**A**) mRNA levels measured by RT-qPCR in HCT116LR cells that stably express NRIP1 (RIP140) or not (GFP). Results are the fold change ± SD relative to control (GFP) after normalization to 28S mRNA. (**B**) Western blot analysis of MSH2 and MSH6 in the same cells described in panel A (left), and data quantification (right) in arbitrary units (AU). (**C**) mRNA levels quantified (as in panel A) in HCT116HR cells transiently transfected with control (siCTRL) and anti-NRIP1 (siNRIP1) siRNAs. *n* = 3 independent experiments for each condition. (**D**) Luciferase activity of HCT116LR cells co-transfected with the *MSH2* or *MSH6* promoter reporter and with increasing concentrations of pEF-cmyc-RIP140. RLU, relative luciferase values (i.e., percentage of the activity measured in the absence of NRIP1). Data are the mean ± SD of 3 independent experiments. (**E**) Schematic representations of the luciferase reporters with mutated Sp1 sites in the MSH2/MSH6 promoters. Lower panel, luciferase activity of HCT116LR cells co-transfected with the indicated mutated reporter constructs and increasing concentrations of pEF-cmyc-RIP140 (mean ± SD of 3 independent experiments). (**F**) ChIP assay using HT29 cells and anti-IgG, anti-H3pan or anti-NRIP1 antibodies. Purified DNA was amplified by qPCR using *MSH6* (2 and 4) or *MSH2* promoter (7) primer pairs (see upper panel in **E**). For all panels: * *p* < 0.05, ** *p* < 0.01 and *** *p* < 0.001 (Mann–Whitney test).

To get closer to the human pathology, we then analyzed the NRIP1 effect on *MutSα* gene expression in human CRC cell lines. *MSH2/MSH6* mRNA (Figure 2A) and protein (Figure 2B) levels were also increased in HCT116 cells (MSI CRC line) that stably overexpress NRIP1 compared with control cells (empty vector). We obtained similar results after transient transfection of HCT116 cells (Supplementary Figure S1A). *MSH2/MSH6* mRNA (Figure 2C) and protein levels (Supplementary Figure S1B) significantly decreased in HCT116 cells upon *NRIP1* silencing. The effect of NRIP1 ectopic expression and silencing were confirmed in other CRC cells, namely SW480 cells (Supplementary Figure S2A,B) and RKO cells (Supplementary Figure S2C,D), respectively. In addition, similar results were obtained in the human HT29 CRC cell line (data not shown).

To investigate the underlying molecular mechanisms, we transiently transfected HCT116 cells with luciferase reporter constructs that harbor the proximal promoter region of *MSH2* or *MSH6*. The luciferase activity driven by the *MSH2* and *MSH6* promoters was significantly increased by NRIP1 transfection in a dose-dependent manner (Figure 2D), supporting a positive transcriptional regulation of these two genes.

Although NRIP1 was first identified as a transcriptional repressor, we and others reported positive regulation of gene expression (for a review see [35]) that, at least in part, implicates Sp1-mediated mechanisms [19]. As both *MSH2* and *MSH6* promoters harbor Sp1 binding sites, we tested whether mutation of these sites affected their transcriptional regulation by NRIP1 (Figure 2E, top). The induction of luciferase activity by NRIP1 was significantly reduced when we used the Sp1-mutated reporter constructs (Figure 2E, bottom panels), suggesting that NRIP1 regulation of *MSH2* and *MSH6* expression is partly Sp1-mediated.

Finally, ChIP experiments demonstrated the specific recruitment of NRIP1 only to the *MSH2* and *MSH6* promoter sequences that encompassed the Sp1-1 and the Sp1-1/Sp1-2 binding sites that conferred regulation by NRIP1 in the luciferase assays (Msh2-7 and Msh6-2 sequences, respectively) (Figure 2F). We did not observe any NRIP1 recruitment to the upstream region encompassing the Sp1-3 to Sp1-5 binding sites (Msh6-4 sequence), whereas significant amplifications were observed after anti-histone H3 ChIP (αH3pan). These results indicated that *MSH2* and *MSH6* are direct transcriptional targets of NRIP1.

### 3.3. Correlations between NRIP1 and MSH2/MSH6 Expression in Human CRC Samples

**Figure 3:**
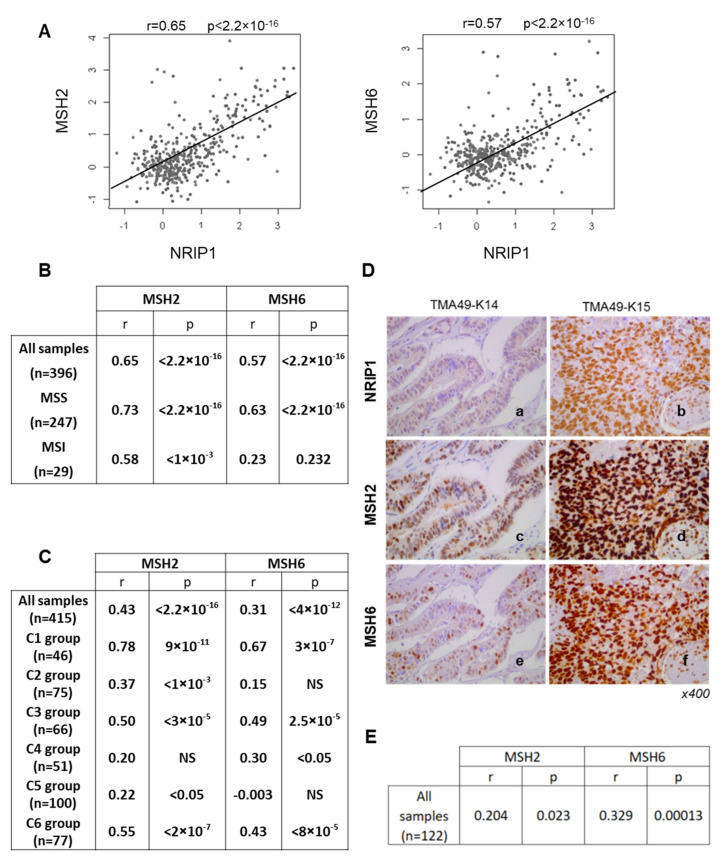
*NRIP1* and *MUTSα* gene expression in human CRC samples. (**A**) Correlation plots between *NRIP1*, *MSH2* (left panel) and *MSH6* (right panel) gene expression in the whole cohort (*n* = 396 CRC samples) [33]. (**B**) Correlations in the whole cohort and in subgroups corresponding to MSS or MSI CRC samples analyzed with the Spearman test. (**C**) Correlation between *NRIP1* and *MSH2/6* mRNA expression in the TCGA-COAD RNA-seq dataset [3] (*n* = 415 CRC samples classified according to their molecular subtypes [34]). (**D**) Representative images showing NRIP1 (**a**,**b**), MSH2 (**c**,**d**) and MSH6 (**e**,**f**) immunostaining in a stage IV primary CRC (**a**,**c**,**e**) and its synchronous liver metastasis (**b**,**d**,**f**). Magnification ×400. (**E**) Correlation between NRIP1 and MSH2/MSH6 protein levels in the CRC samples described in Supplementary Table S2 and illustrated in panel E. The Spearman correlation coefficient and *p*-values are indicated.

To validate these data in human CRC, we analyzed three transcriptomic datasets. Analysis of the data from 396 CRC tissue samples [33] showed that *NRIP1* mRNA levels were significantly correlated with *MSH2* (ρ = 0.65; *p* < 2.2 × 10^−16^) and *MSH6* (ρ = 0.57; *p* < 2.2 × 10^−16^) mRNA levels (Figure 3A,B). The correlations were stronger when we considered only MSS CRC samples, although the correlation with *MSH2* was also significant in MSI tumors despite the low number of samples (Figure 3B).

Analysis of the TCGA-COAD RNA-seq dataset (*n* = 415 CRC samples) [3] confirmed the significant correlation between *NRIP1* and *MSH2* (r = 0.43; *p* < 2.2 × 10^−16^) and *MSH6* (r = 0.31; *p* = 3.6 × 10^−16^) mRNA levels (Figure 3C). We then considered the six different CRC molecular subtypes previously identified [34], C1 (immune pathways down regulated), C2 (enriched in dMMR tumors), C3 (KRAS mutated), C4 (enriched in cancer stem cells), C5 (wnt signaling up) and C6 (enriched for tumors with a normal-like gene expression profile). We observed the best correlations in the C1, C3 and C6 subgroups. Conversely, correlations were not significant in the C4 (for *MSH2*) and in the C2 and C5 groups (for *MSH6*) (Figure 3C). A significant positive correlation between *NRIP1* and *MSH2* gene expression (data not shown) was also found in a third dataset corresponding to a multicenter cohort of 750 patients set up by the French national CIT program [34]. Finally, we confirmed the significant associations between NRIP1 and MSH2 (*p* = 0.023) and MSH6 (*p* < 0.00013) also by analysis of their protein level by IHC in 122 CRC samples (Figure 3D,E, and Supplementary Table S2). Altogether, these data strongly supported the positive regulation of MSH2 and MSH6 gene/protein expression by NRIP1 in human CRC.

### 3.4. Functional Consequences of MutSα Regulation by NRIP1

**Figure 4:**
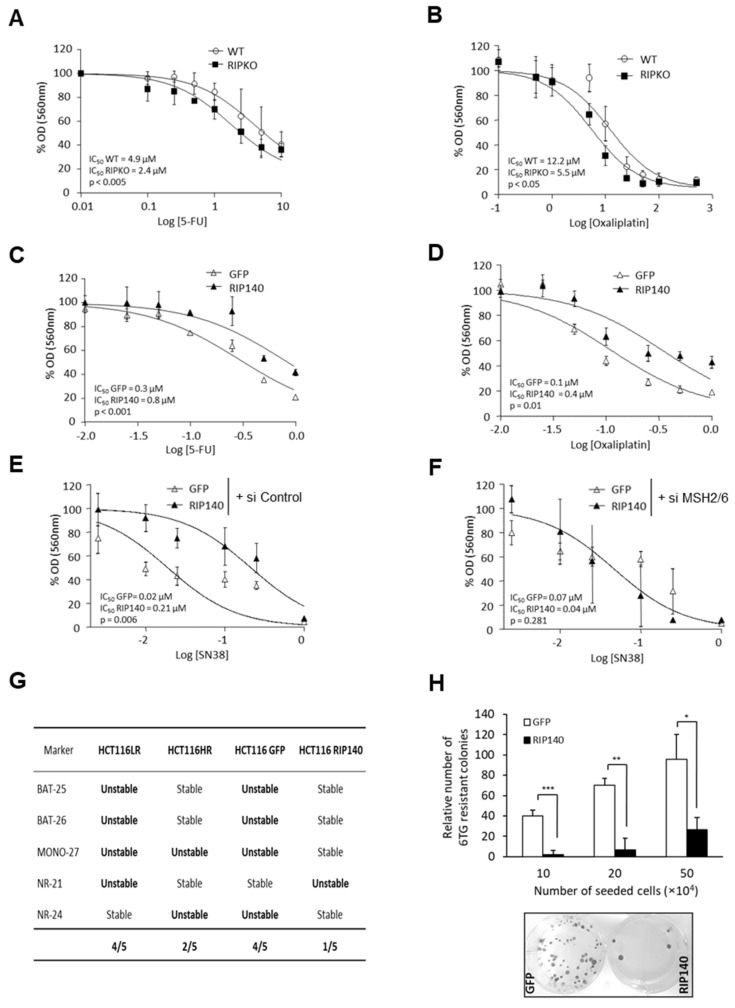
Cellular consequences of MutSα regulation by NRIP1. (**A**,**B**) Cytotoxic effect of increasing doses of 5-fluorouracil (5-FU) (panel **A**) and oxaliplatin (panel **B**) in WT and RIPKO MEFs; the IC_50_ and *p* values are indicated. (**C**,**D**) Same experiment in HCT116-RIP140 (RIP140) or HCT116-GFP (GFP) for 5-FU (panel **C**) and oxaliplatin (panel **D**). (**E**,**F**) SN38 effect in HCT116-GFP and HCT116-RIP140 cells transfected with control siRNA (panel **E**) or with siRNAs targeting *MSH2* and *MSH6* (panel **F**). (**G**) MSI status of the indicated cell lines (raw data in Supplementary Figure S5B,C). (**H**) HPRT mutation assay after exposure to 6-TG of HCT116-GFP or HCT116-RIP140 (three seeding conditions). Results are the mean ± SD of triplicates for each condition. * *p* < 0.05, ** *p* < 0.01 and *** *p* < 0.001.

One of the cellular consequences of disrupting the MMR system is a differential sensitivity to cytotoxic drugs used in CRC chemotherapy such as 5-FU [36]. Using our cell models (HCT116 cells and MEFs), we observed higher sensitivity to 5-FU, oxaliplatin and SN38 (the active metabolite of irinotecan) in RIPKO than in WT MEFs (Figure 4A,B; Supplementary Figure S3 and Table S3). This sensitivity was significantly decreased upon NRIP1 overexpression in HCT116 cells (Figure 4C–E; Supplementary Table S3). To determine whether the difference in drug sensitivity was dependent on *MSH2* and *MSH6* gene expression induction, we monitored the response to SN38 in HCT116 cells that overexpress or not NRIP1 after siRNA-mediated knockdown of both *MSH2* and *MSH6.* The specificity and efficacy of the two siRNAs were validated (Supplementary Figure S4). *MSH2* and *MSH6* downregulation completely abolished the effect of NRIP1 overexpression on sensitivity to SN38 (Figure 4F). This demonstrated that the NRIP1 effect on SN38 sensitivity was dependent on its regulation of *MutSα* gene expression.

As MSI is the major consequence of dMMR [37], we asked whether *MSH2* and *MSH6* expression regulation by NRIP1 could affect MSI in human CRC cells by analyzing five nearly monomorphic mononucleotide repeat markers (BAT-25, BAT-26, MONO-27, NR-21, NR-24) using a multiplexed PCR assay. First, we analyzed two HCT116 cell lines with low (HCT116LR) and high (HCT116HR) NRIP1 expression levels, respectively. As expected, *MSH2* and *MSH6* expression levels were higher in HCT116HR than in HCT116LR cells (Supplementary Figure S5A). Microsatellite analysis revealed higher instability in HCT116LR cells. Indeed, four of the five mononucleotide repeat loci were unstable in HCT116LR cells and only two in HCT116HR cells (Figure 4G and Supplementary Figure S5B).

To demonstrate clearly that NRIP1 differential expression was the cause of this instability, we performed the same analysis in HCT116LR cells after transfection of NRIP1 (HCT116-RIP140 cells) and found that they were more stable than cells transfected with the empty vector (HCT116-GFP) (Figure 4G and Supplementary Figure S5C). These findings indicated that high NRIP1 levels correlated with high *MSH2* and *MSH6* gene expression and with lower MSI.

To validate the consequences of MutSα regulation by NRIP1 functionally, we performed a HPRT mutation assay in HCT116 cells that overexpress or not NRIP1. MMR activity is required to repair O6-methylguanine lesions induced by MNNG [38] that can be detected in the *HPRT* gene using a colony formation assay in the presence of 6-TG. The number of 6-TG resistant colonies was strongly and significantly decreased in cells that overexpress NRIP1 (Figure 4H), thus validating our observation that NRIP1 positively regulates MMR activity.

All these data indicated that *MSH2* and *MSH6* expression regulation by NRIP1 correlated with changes in drug sensitivity, MSI and mutation repair. Therefore, we hypothesized that MSI-induced NRIP1 loss-of-function mutations might lead to decreased expression of genes coding for MutSα and might strongly amplify MSI and contribute to intestinal tumorigenesis.

### 3.5. Identification and Characterization of a NRIP1 Frameshift Mutation

To test this hypothesis, we screened several MSI CRC cell lines by sequencing the whole *NRIP1* coding exon and identified in LoVo cells a frameshift mutation that leads to the deletion of an adenosine nucleotide (c.2184delA; p.E729fs) and to a truncated protein that lacks the last 431 amino acids and exhibits a short specific RKLP sequence (Supplementary Figure S6A).

**Figure 5:**
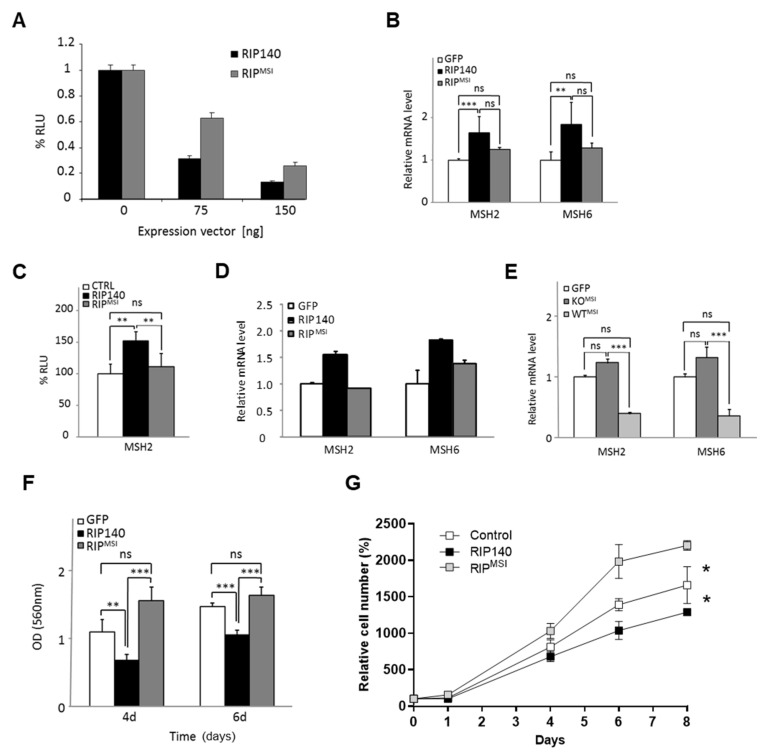
Biological characterization of the RIP^MSI^ frameshift mutation. (**A**) Intrinsic transrepression assay in HCT116LR cells transiently transfected with increasing doses of the Gal4DBD-fused NRIP1 and RIP^MSI^ expression vectors. (**B**) *MSH2* and *MSH6* mRNA levels in RIPKO MEFs stably expressing GFP, GFP-RIP140 or GFP-RIP^MSI^; *n* = 3 independent experiments. (**C**) Luciferase assay with the MSH2 gene reporter and increasing doses of NRIP1 or RIP^MSI^ expression vectors (mean ± SD; *n* = 3 independent experiments). (**D**) *MSH2* and *MSH6* mRNA levels in HT29 cells transiently transfected with pEGFP, pEGFP-RIP140 or pEGFP-RIP^MSI^ expression vectors. (**E**) *MSH2* and *MSH6* mRNA levels in stably transfected MEF-WT^MSI^ and MEF-KO^MSI^. Data were normalized as compared to WT and RIPKO control cells expressing GFP alone and set at 1. (**F**) Cell proliferation of RIPKO MEFs stably expressing GFP, GFP-RIP140 or GFP-RIP^MSI^ at day 4 and 6 after seeding. Results are the fold-change vs. control (GFP) cells after normalization to the cell density at day 1; *n* = 3 independent experiments. (**G**) Cell proliferation of human RKO CRC cells transiently transfected with pEGFP, pEGFP-RIP140 or pEGFP-RIP^MSI^ expression vectors over 8 days. Results are the fold-change (triplicates) after normalization to the cell density at day 0. For all panels: data are the mean ± SD; ns = not significant, * *p* < 0.05, ** *p* < 0.01, *** *p* < 0.001 (Kruskal–Wallis test).

This NRIP1 variant (called RIP^MSI^) displayed the same punctuate subcellular localization as the WT protein, after transient transfection of HCT116 cells (Supplementary Figure S6B). However, in reporter assays using Gal4DBD constructs in which the NRIP1 and RIP^MSI^ coding sequences were fused to the DNA binding domain of the Gal4 transcription factor, we observed a clear decrease in the intrinsic transrepression of RIP^MSI^ (Figure 5A), probably due to the loss of the RD3 and RD4 repression domains (Supplementary Figure S6A).

Analysis of RIP^MSI^ biological activity in RIPKO MEFs that stably express NRIP1 or RIP^MSI^ (Supplementary Figure S7A) showed that *MSH2* and *MSH6* gene expression was increased less efficiently by RIP^MSI^ than NRIP1 (Figure 5B and Supplementary Figure S7B). In luciferase reporter assays (Figure 5C), *MSH2* gene promoter activity was lower in cells transfected with RIP^MSI^ than NRIP1. Similarly, endogenous *MSH2* and *MSH6* upregulation in HT29 CRC cells was lower after transient overexpression of RIP^MSI^ than NRIP1 (Figure 5D and Supplementary Figure S7C). Interestingly, RIP^MSI^ seemed to exert a dominant negative effect because *MSH2* and *MSH6* mRNA and protein expression were slightly increased in RIPKO MEFs (Figure 5E and Supplementary Figure S8A) and reduced in WT MEFs upon transfection with RIP^MSI^ (see Supplementary Figure S8B).

We observed the same dominant negative effect on the 5-FU response with an increased sensitivity of RIP^MSI^-expressing cells compared to decreased sensitivity of NRIP1-expressing cells (Supplementary Figure S8C). Finally, ectopic expression of RIP^MSI^ resulted in an increase in RIPKO MEF proliferation compared with the significant inhibition observed with NRIP1 (Figure 5F). We obtained similar data in CRC cell lines (Figure 5G and data not shown). This suggested that RIP^MSI^ abrogates NRIP1 anti-proliferative functions in a dominant-negative manner. The expression of this MSI-target mutant may play a critical role in intestinal tumorigenesis and might represent a major prognosis determinant in MSI CRC.

### 3.6. RIP^MSI^ in MSI CRC Clinical Samples

We first investigated the presence of the RIP^MSI^ mutation in 194 non-metastatic MSI CRC samples (see Table 1 for patients and tumor characteristics). We detected RIP^MSI^ in 22.2% of samples. RIP^MSI^ frequency was not correlated with any clinical or molecular characteristic, but was much lower in stage III tumors (14.3%) than in stage I and II tumors (33.3 and 24.8%, respectively). We found RIP^MSI^ in 23.8% of cancers in the subgroup of dMLH1 CRCs (i.e., sporadic cases with *MLH1* promoter hypermethylation or LS cases linked to a *MLH1* gene mutation). In this subgroup, RIP^MSI^ frequency in stage III tumors was even lower (12.2% vs. 27.3 and 30% in stage I and II tumors, respectively; *p* = 0.038).

**Figure 6:**
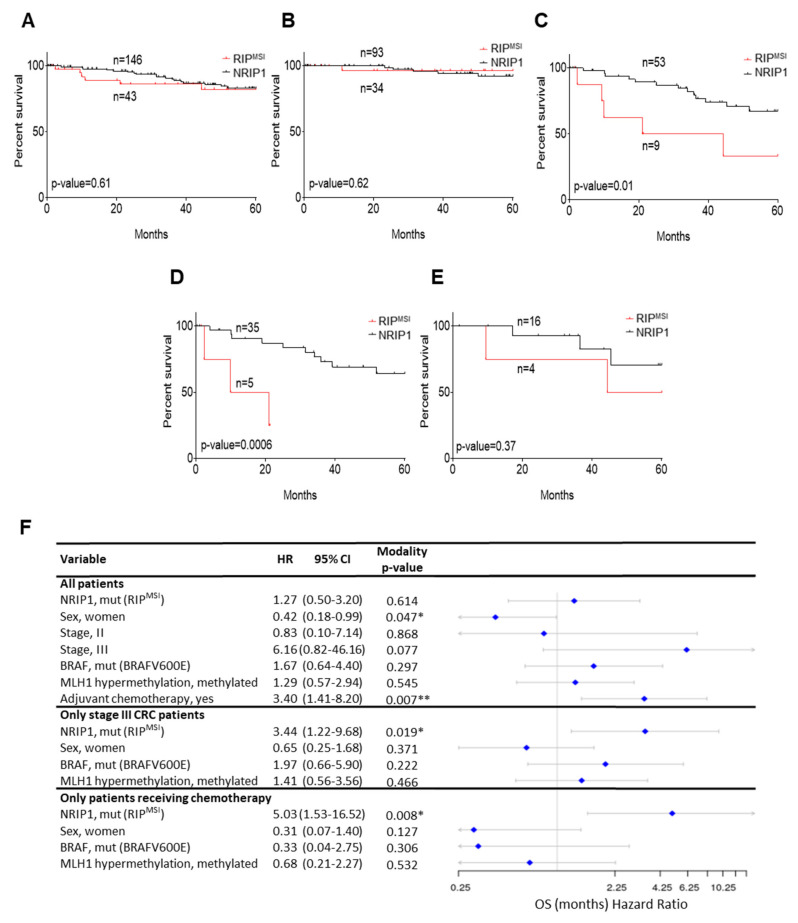
RIP^MSI^ frameshift mutation and survival of patients with CRC. Kaplan–Meier plots of OS rate in patients with CRC divided according to the presence/absence of the RIP^MSI^ mutation in the tumor DNA. (**A**) Whole cohort (*n* = 189); Patients with (**B**) stage I/II CRC (*n* = 127); (**C**) stage III CRC (*n* = 62); (**D**) stage III CRC with dMLH1 (*n* = 40); (**E**) stage III CRC and proficient MLH1 (*n* = 20). (**F**) Forest plot of the associations between the indicated variables and OS (Cox univariate analysis; * *p* < 0.05 and ** *p* < 0.01) in the whole cohort and in the indicated subgroups. HR, Cox Hazard Ratio; CI, Confidence Interval of HR.

We then analyzed OS in the function of RIP^MSI^ presence. The OS rate was not different between patients with CRC harboring or not the RIP^MSI^ mutation in the whole cohort (Figure 6A) and in the group with stage I/II CRCs (Figure 6B). Conversely, the OS rate was significantly lower in patients with stage III CRC harboring the RIP^MSI^ mutation (*p* = 0.01) (Figure 6C). As our molecular data linked NRIP1 to MutSα gene expression, we expected a stronger impact of RIP^MSI^ in patients with dMLH1 CRC (i.e., tumors deficient for MutL, the other component of the MMR system; additive effect of the two alterations). Indeed, the OS rate was lower in patients with dMLH1 stage III CRC harboring also the RIP^MSI^ mutation (*p* = 0.0006) (Figure 6D), whereas it was comparable in patients with MLH1-proficient tumors with/without the RIP^MSI^ mutation (Figure 6E). Due to the low number of RIP^MSI^ mutated samples, this difference between MLH1 proficient and deficient tumors, although highly significant, needs to be validated in further studies. The RIP^MSI^ mutation was also significantly associated with poor prognosis in patients with stage III CRC and (i) hypermethylated MLH1 (sporadic forms), which represents the most frequent dMLH1 CRCs (*p* < 0.0001, Supplementary Figure S9A); (ii) harboring the BRAFV600E mutation (*p* = 0.0019, Supplementary Figure S9B) and (iii) older than 55 years (*p* < 0.0001, Supplementary Figure S9C). These features correspond mainly to patients with sporadic MSI CRC. Conversely, OS of stage III CRC was not influenced by the RIP^MSI^ mutation in patients with familial CRC, wild-type BRAF tumors or younger than 55 years (Supplementary Figure S9D–F). Finally, the OS rate was lower also in patients with RIP^MSI^ stage III CRC treated by chemotherapy (Supplementary Figure S9G, *p* = 0.003), but not in untreated patients (Supplementary Figure S9H, *p* = 0.89).

In univariate Cox analyses, only sex and treatment were significantly associated with OS in the whole cohort (Figure 6F), whereas the RIP^MSI^ mutation was significantly correlated with OS in patients with stage III CRC and in those receiving chemotherapy (*p* = 0.019 and 0.008, respectively). Altogether, the RIP^MSI^ mutation was associated with decreased OS in patients with advanced CRC, especially in patients with dMLH1 or treated with chemotherapy.

## 4. Discussion

CRC is a frequent malignancy displaying MSI due to MMR defects. We recently showed that the transcription factor NRIP1 has an important role in the regulation of intestinal homeostasis and tumorigenesis [23]. Here, we demonstrated: (1) NRIP1’s role in the control of MutSα gene expression and MSI in intestinal epithelial cells, and (2) the prognostic value of a truncated NRIP1 variant in MSI CRC.

First, our data clearly demonstrated that NRIP1 positively regulates *MSH2* and *MSH6* expression in mouse and human cells and tissues. This regulation takes place at the transcriptional level and is at least partly Sp1-mediated. E2F factors [39] and p53 [40] also regulate the expression of these two genes and might also mediate the positive regulation by NRIP1. Interestingly, the transcriptional deregulation of the *MSH6* and/or *MSH2* genes by BCL2 [41] or HIF1α [28] was shown to induce MSI. Recently, Fang et al. showed that CRTC2 acts as a tumor suppressor gene involved in genomic integrity by inducing *MSH6* gene transcription [42]. Therefore, besides genetic and epigenetic modifications, MSI may result from MMR gene transcriptional deregulations. Our findings support these observations by linking the regulation of *MutSα* gene expression by NRIP1 to a lower degree of MSI.

Analysis of previously published transcriptomic data [34] in the function of the CRC molecular classification [43] further strengthened our conclusions. Indeed, *NRIP1* expression was significantly lower in the CMS1 subgroup, which corresponds to MSI CRCs (Supplementary Figure S10). We previously described NRIP1 as a regulator of the Wnt signaling pathway in CRC [23] and also as a major metabolic regulator [44]. Because of these pleiotropic actions, NRIP1 might be at the crossroads of different consensus clusters, including CMS1 (MSI), CMS2 (Wnt activation) and CMS3 (metabolic deregulation). We could hypothesize that non-consensus tumors (around 20% of the samples), which do not have a consistent subtype profile, might correspond to NRIP1-mutated tumors, thus explaining the difficulty to classify them in a single CMS.

In addition to MSI, MMR activity tightly regulates other cellular parameters [45]. In agreement, we demonstrated that NRIP1 modulates the sensitivity to the SN38 cytotoxic drug through the regulation of *MSH2* and *MSH6* expression, and influences the mutator phenotype. Concerning the effects of NRIP1 on cell sensitivity to 5FU, our data did not fit with (a) MMR-mediated mechanism(s) suggesting that other cellular pathways involved in 5FU resistance (see Blondy et al. [46] for a recent review) could be regulated by NRIP1. Other phenotypic consequences, such as chromosomal abnormalities or centrosome amplification, are linked to altered MMR activity [47], and additional work is necessary to elucidate the precise NRIP1 role on such parameters. Regarding cancer development and progression, MMR deficiency increases mutation frequency, leading to malignancy [48]. In MSI CRC cells and samples, we detected a NRIP1 frameshift mutation at the heterozygous status (RIP^MSI^). Several findings then suggested that the RIP^MSI^ mutation exerts a dominant negative effect on MMR gene expression regulation, drug response and, importantly, inhibition of cell proliferation. Of note, in a family of patients with congenital anomalies of kidneys and the urinary tract, a heterozygous truncating mutation in the *NRIP1* gene [49] acts in a dominant manner and interferes with the retinoic acid cellular pathway.

In full agreement with our molecular and cellular data, our survival results suggest that the poor prognosis conferred by the RIP^MSI^ mutation is more significant in the context of MLH1 deficiency. Indeed, as expected from the positive effect of NRIP1 on MutSα expression, dMLH1 tumors harboring the RIP^MSI^ mutation displayed a more severe defect of MMR activity, resulting from the combination of MutL and MutS alterations. Epigenetic silencing of the *MLH1* gene explains almost all sporadic MSI CRC, and germline *MLH1* mutations cause half of the cases of LS-related MSI CRC. More than 30 years ago, it was proposed that a cascade of alterations in genes controlling MSI might unfold genetic instability during tumor progression [50]. The *NRIP1* gene is clearly involved in this MSI amplification loop, and dMLH1 tumors harboring the RIP^MSI^ mutation may not only exhibit a higher overall mutation rate but also a different spectrum of mutations.

Importantly, the RIP^MSI^ mutation is associated with decreased OS in patients with stage III MSI CRC. Despite the significant progress in CRC treatment, patient outcome is still difficult to predict. Physicians lack robust prognostic biomarkers for patient management and follow-up after curative resection. Many studies assessed the potential prognostic impact of several somatic mutations (e.g., BRAF, KRAS and TP53 mutations), but none has been validated as a reproducible prognostic biomarker for MSI CRC. RIP^MSI^ is the first mutation that might improve disease prognostication for MSI tumors. More work is required to confirm that the RIP^MSI^ mutation is a predictive biomarker of the response to chemotherapy. Nonetheless, its detection may allow classifying more precisely patients at high risk to optimize their management (e.g., more intense chemotherapy protocols or early treatment with immune checkpoint inhibitors). Conversely, in early-stage CRC, the RIP^MSI^ mutation might contribute to the good prognostic value of MSI, probably by promoting a better immune response [11]. During tumor progression, this beneficial effect is lost when tumors escape the immune surveillance. The association of the RIP^MSI^ mutation with poor prognosis in advanced cases might reflect its dominant positive activity on cell proliferation.

## 5. Conclusions

In conclusion, we identified the *NRIP1* gene both as a MSI amplifier and as a mutation target in MSI CRC. The RIP^MSI^ variant, which acts in a dominant-negative manner, appears to be a key event in MSI-driven tumorigenesis and might be a major determinant of prognosis and treatment response in MSI CRC.

## Figures and Tables

**Table 1:** Patients and tumor characteristics. Patients’ clinical features and anatomopathological characteristics of the related CRC samples used for the analysis of the RIP^MSI^ mutation (all patients and only patients with dMLH1 CRC). The mutation frequency in each group is indicated.

	All CRCs *n* = 194		dMLH1 CRCs *n* = 122	
Characteristic	Population *n* (%)	RIP^MSI^ *n* (%)	Population *n* (%)	RIP^MSI^ *n* (%)
NRIP1				
Wild-type	151 (77.8)	/	93 (76.2)	/
Mutated	43 (22.2)	/	29 (23.8)	/
Age				
Age at diagnosis	71.5		77	
(median, years)	[20.7–97]		[20.7–97]	
<55	68 (35.1)	12 (17.6)	28 (23)	6 (21.4)
≥55	123 (63.4)	31 (25.2)	92 (75.4)	23 (25)
Missing data	3 (1.5)	0 (0)	2 (1.6)	0 (0)
Sex				
Men	94 (48.5)	20 (21.3)	53 (43.4)	12 (22.6)
Women	100 (51.5)	23 (23)	69 (56.6)	17 (24.6)
Tumor site				
Colon	171 (88.1)	37 (21.6)	112 (91.8)	26 (23.2)
Rectum	21 (10.2)	6 (28.6)	8 (6.6)	3 (37.5)
Missing data	2 (1)	0 (0)	2 (1.6)	0 (0)
pTNM stage				
I	18 (9.3)	6 (33.3)	11 (9)	3 (27.3)
II	113 (58.2)	28 (24.8)	70 (57.4)	21 (30)
III	63 (32.5)	9 (14.3)	41 (33.6)	5 (12.2) * ^$^
BRAF V600E				
No	91 (46.9)	17 (18.7)	50 (41)	9 (18)
Yes	47 (24.2)	14 (29.8)	47 (38.5)	14 (29.8)
Missing data	56 (28.9)	12 (21.4)	25 (20.5)	6 (24)
MLH1 status				
dMLH1	122 (62.9)	29 (23.8)	/	/
pMLH1	66 (34)	14 (21.2)	/	/
Missing data	6 (3.1)	0 (0)	/	/

* stage III versus stage II: *p* = 0.038. ^$^ stage III versus stage I and II: *p* = 0.042.

## Data Availability

The data presented in this study are openly available in the NCBI Gene Expression Omnibus (http://www.ncbi.nlm.nih.gov/geo/; reference GSE39582, GSE5206 and GSE10402), the NCI Genomic Data Commons (https://gdc.cancer.gov/; reference TCGA-COAD) and in ArrayExpress (https://www.ebi.ac.uk/arrayexpress/; reference MEXP-1245).

## Supplementary Materials

The following are available online at https://www.mdpi.com/article/10.3390/cancers13174449/s1, Figure S1: NRIP1 regulates *MSH2/6* gene expression in transiently transfected HCT116 cells, Figure S2: Regulation of *MSH2/6* gene expression by NRIP1 in other CRC cell lines, Figure S3: NRIP1 affects the response to cytotoxic drugs, Figure S4: Validation of MSH2/MSH6 gene knock-down, Figure S5: *MSH2* and *MSH6* gene expression in the HCT116 cells used to monitor MSI, Figure S6: Subcellular localization of the NRIP1 frameshift mutation, Figure S7: Biological characterization of the NRIP1 frameshift mutation, Figure S8: Effect of the NRIP1 frameshift mutation on MSH2 and MSH6 expression and 5-fluorouracil cytotoxic effect, Figure S9: Prognostic value of the RIPMSI frameshift mutation in different subgroups of patients with MSI CRC, Figure S10: *NRIP1* expression in the different CRC consensus molecular subtypes, Table S1: Sequences of the primers used in qPCR experiments, ChIP assay, next-generation sequencing and siRNA-mediated knock-down, Table S2: Clinical features and anatomopathological characteristics of the related CRC samples used for IHC analysis (*n* = 122 samples from 106 patients), Table S3: Summary of NRIP1 effects on the response to cytotoxic drugs in MEFs and HCT116 cells (IC50 ratio and *p* values are shown), Table S4: Patient and tumor characteristics.
