# Supplementary material for: A Truncated NRIP1 Mutant Amplifies Microsatellite Instability of Colorectal Cancer by Regulating MSH2/MSH6 Expression, and Is a Prognostic Marker of Stage III Tumors
Source: Cancers (Basel). 2021 Sep 3;13(17):4449. doi: 10.3390/cancers13174449 (PMC8430632; doi:10.3390/cancers13174449)
Supplement: Supplementary file 1 [file cancers-13-04449-s001.zip › cancers-1307831-supplementary.pdf]

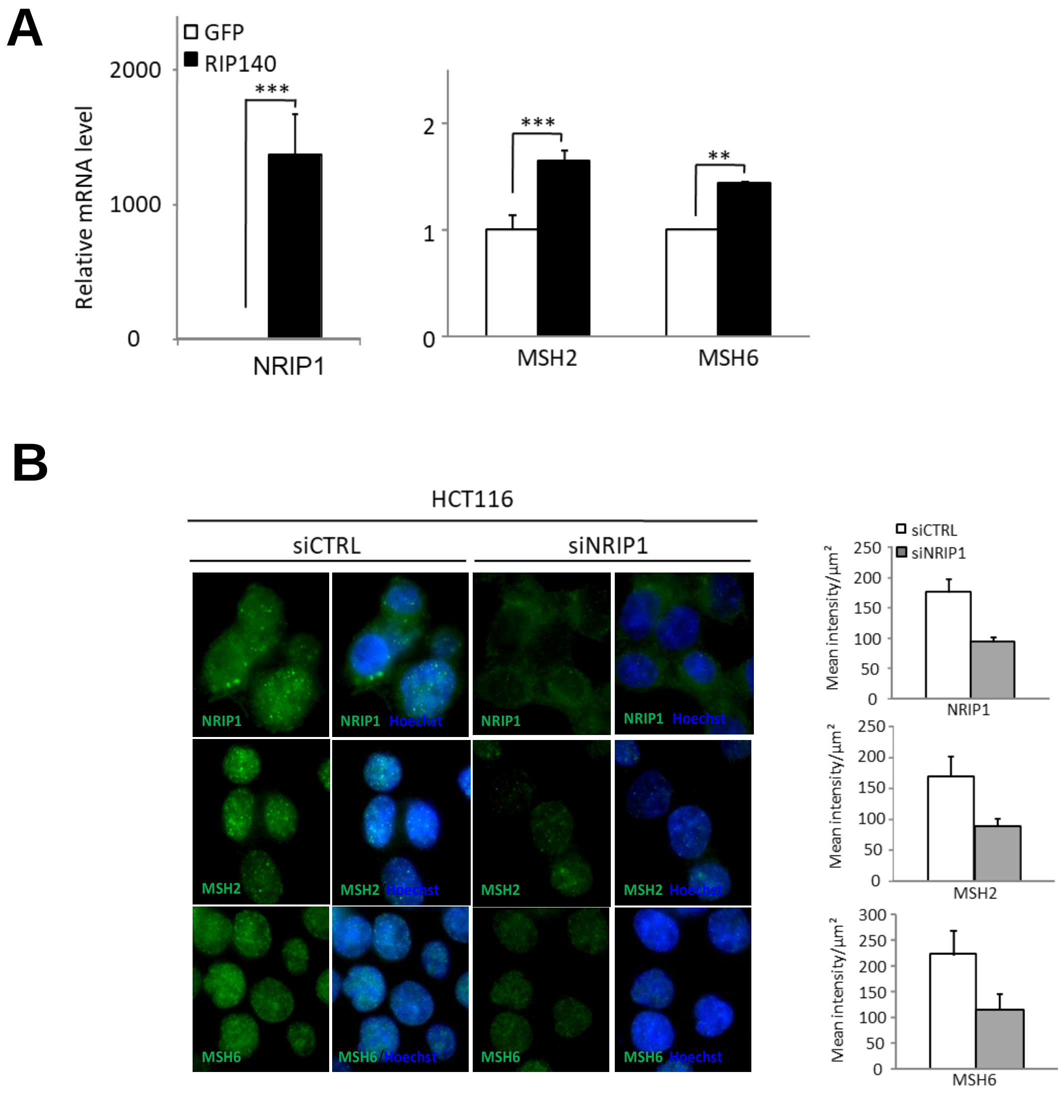

NRIP1 regulates MSH2/6 expression in transiently transfected HCT116 CRC cells

Figure S1

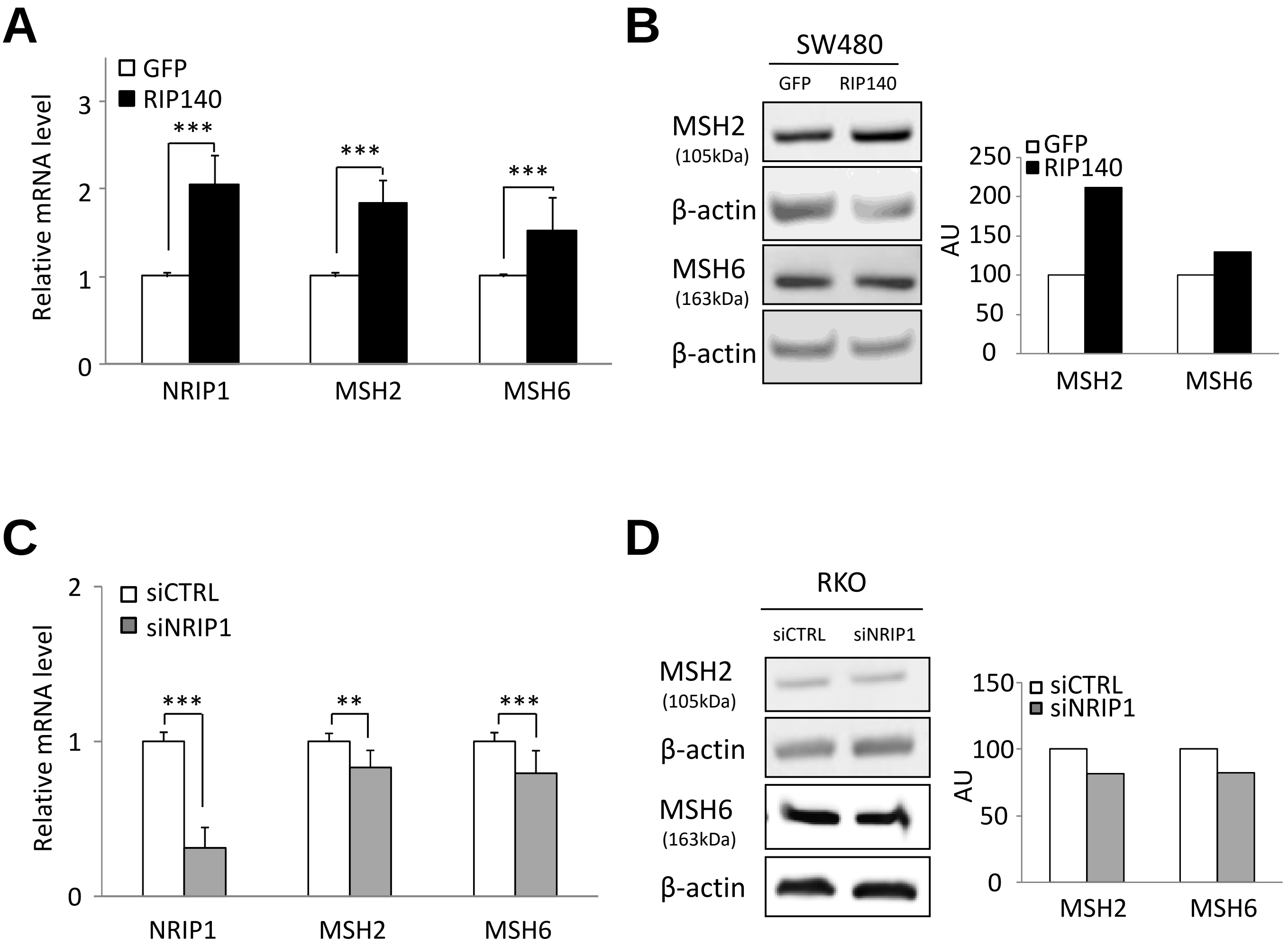

NRIP1 regulates MSH2/6 expression in other CRC cells

Figure S2

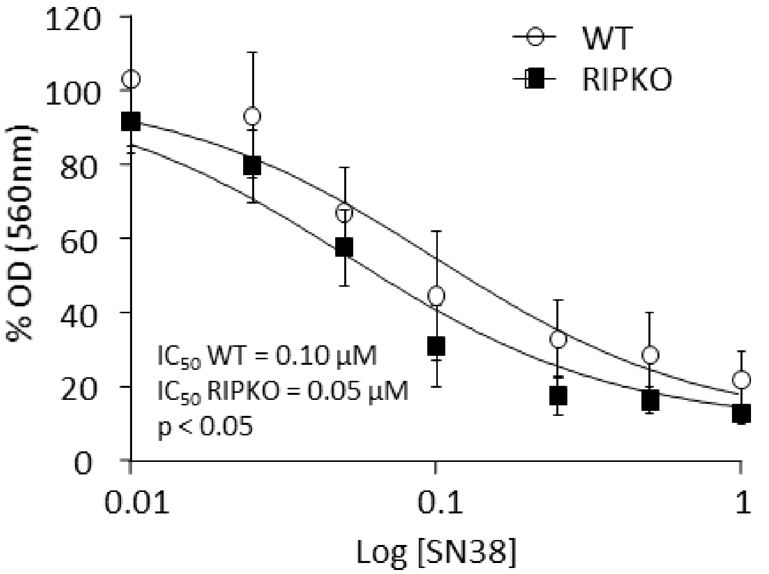

NRIP1 affects the response to SN38 in MEFs

Figure S3

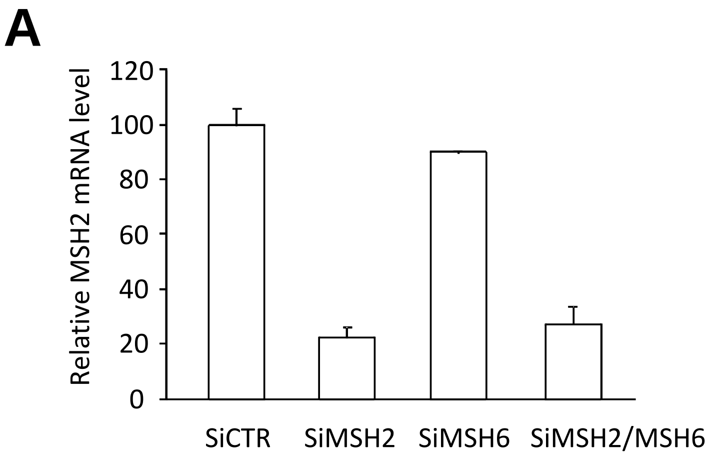

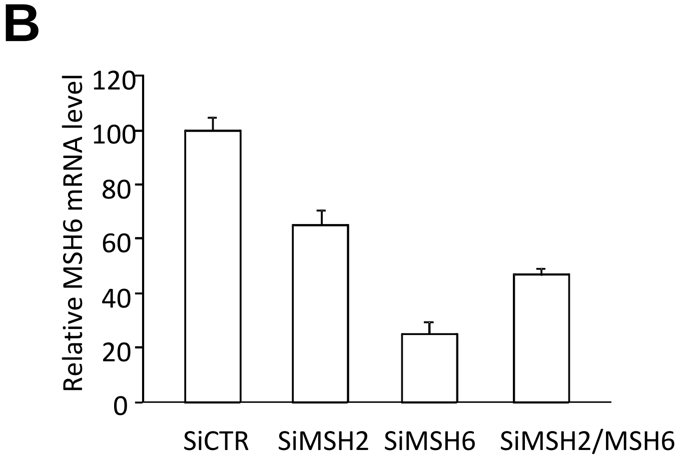

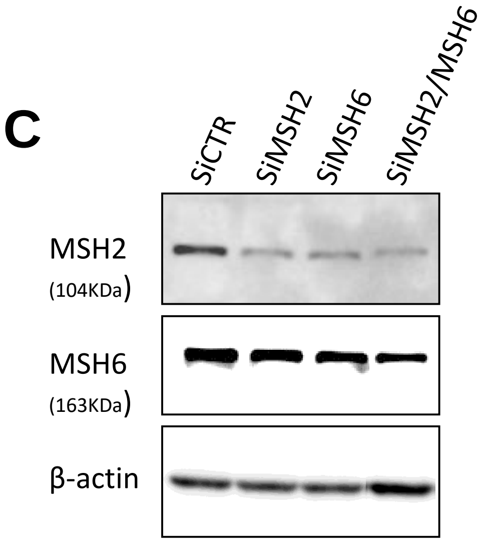

Validation of MSH2/MSH6 gene knock-down

Figure S4

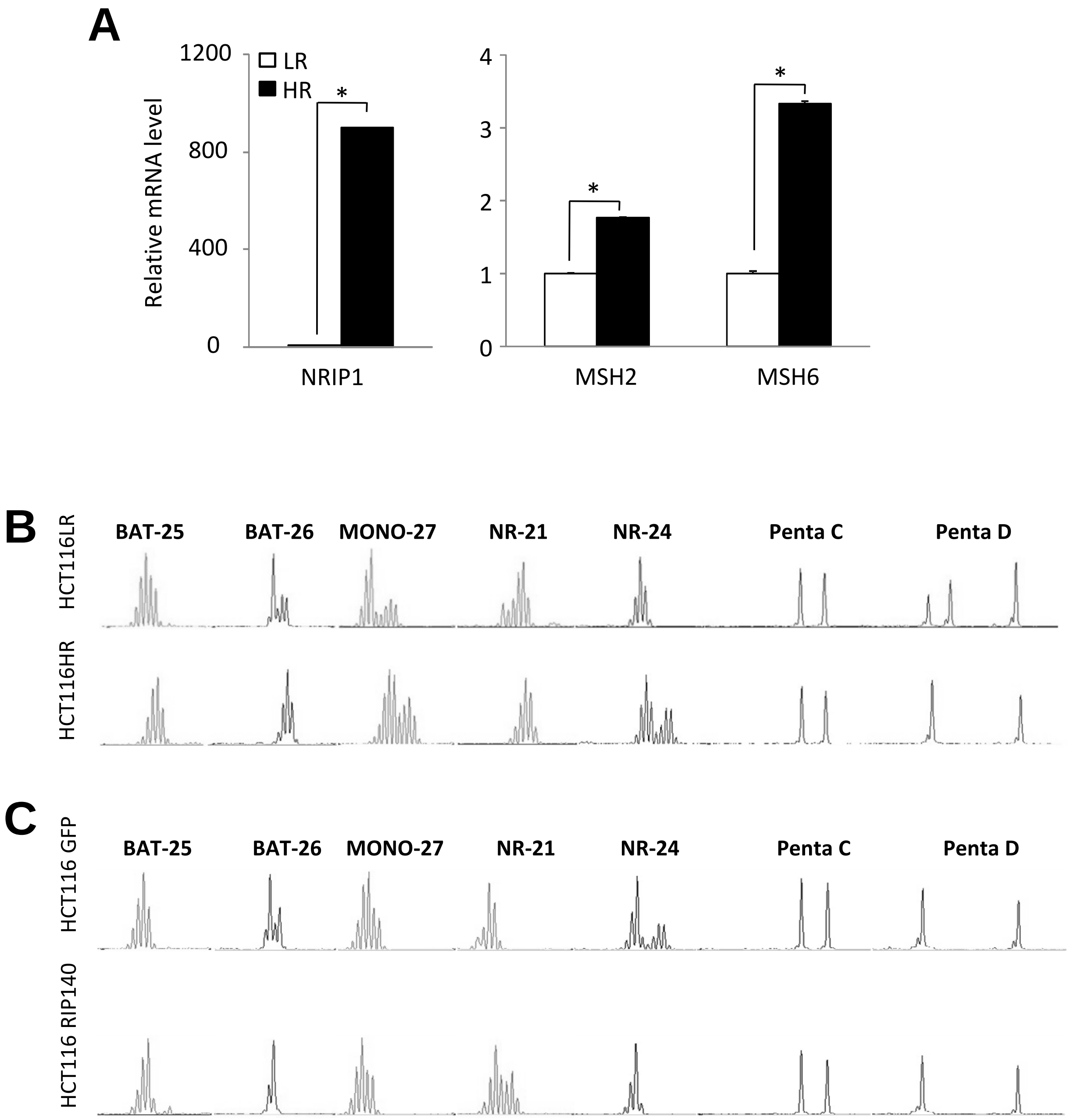

MSH2 and MSH6 gene expression in the HCT116 cells used to monitor MSI

Figure S5

A

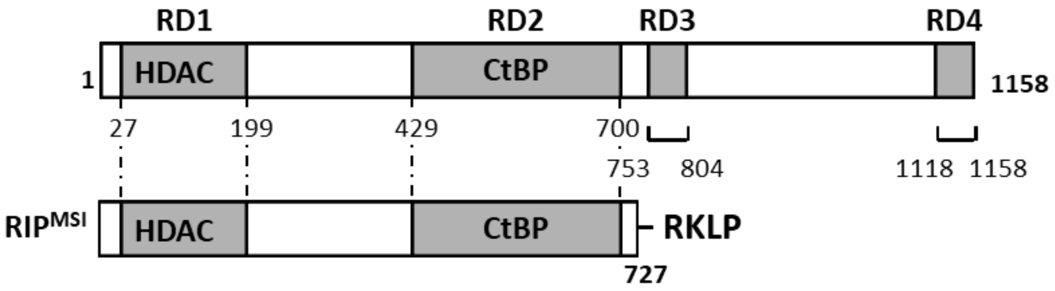

B

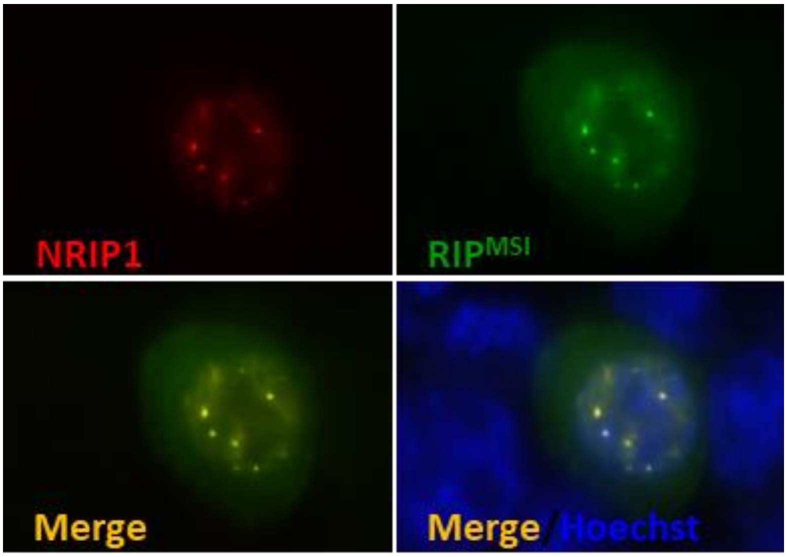

Subcellular localization of the RIP^{MSI} mutation

Figure S6

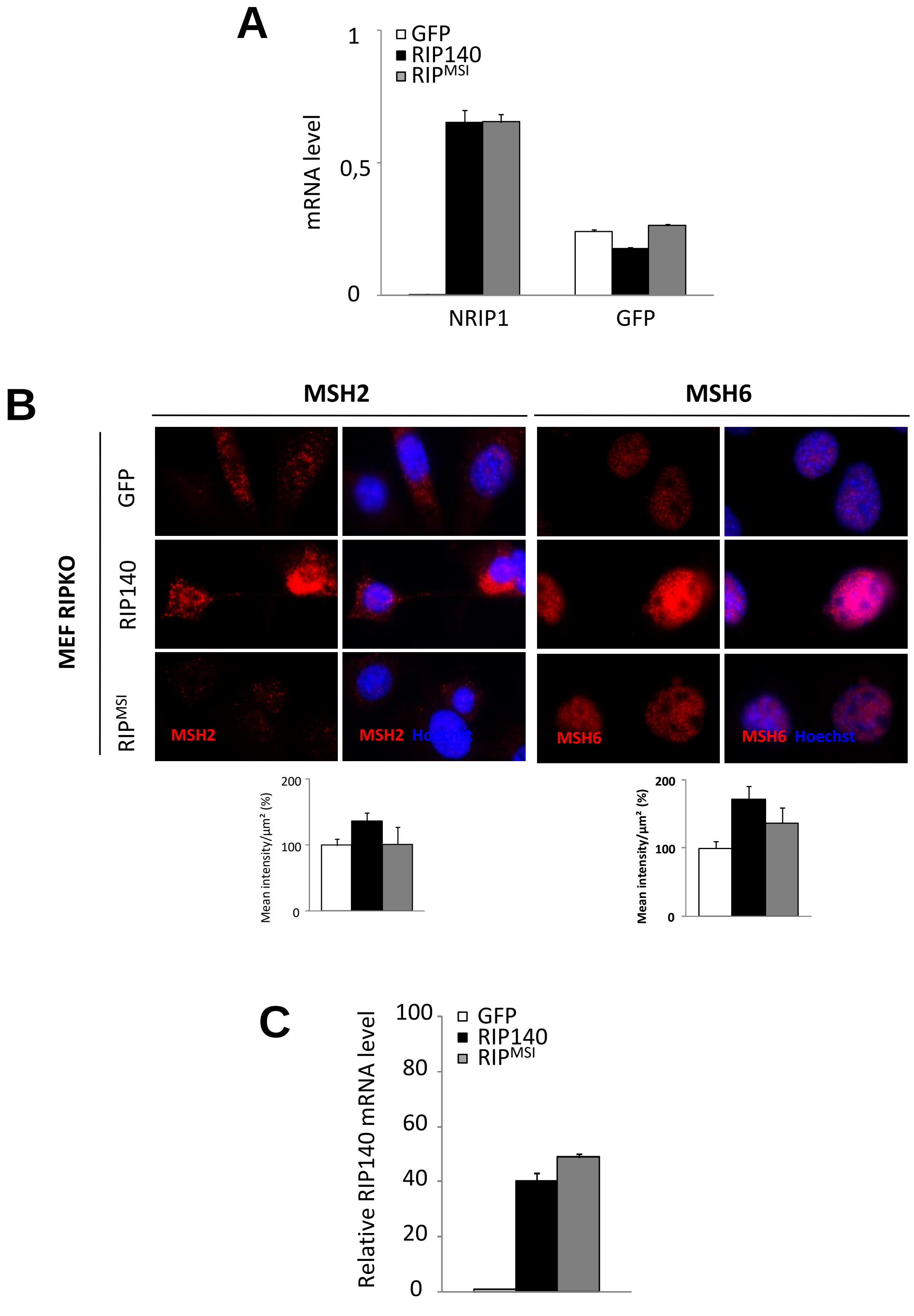

Biological characterization of the RIP^{MSI} mutation

Figure S7

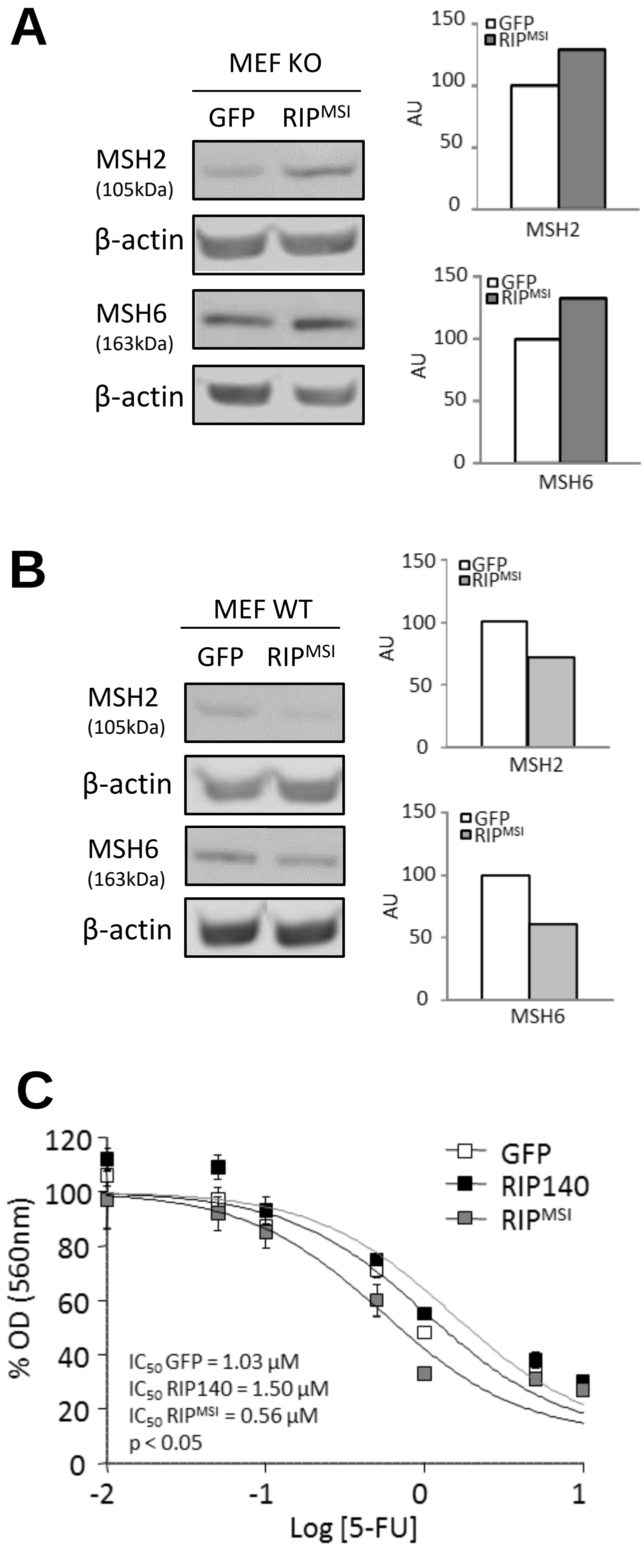

Effect of the RIP^{MSI} mutation on MSH2 and MSH6 expression and 5-FU cytotoxic effect

Figure S8

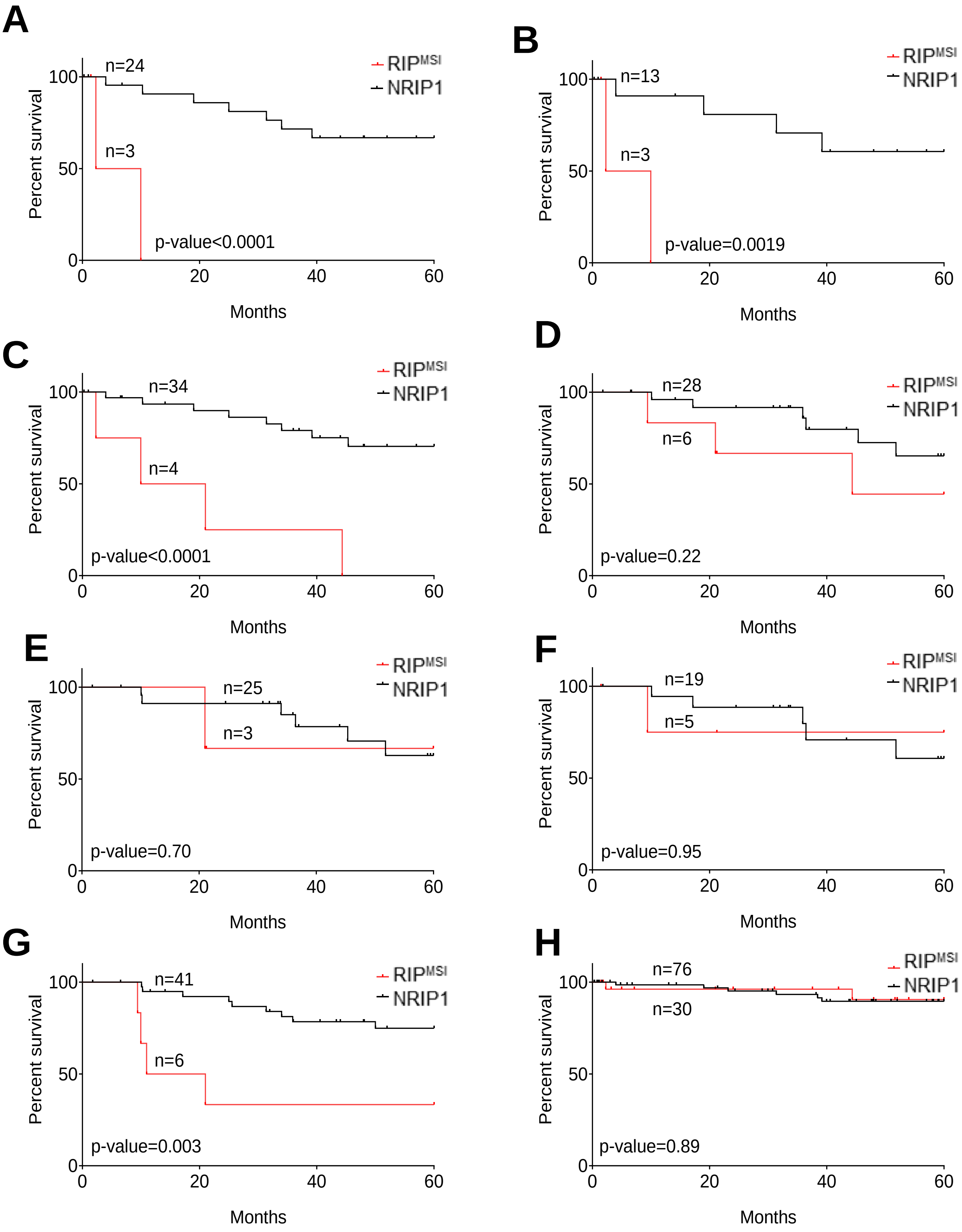

Prognostic value of the RIP^{MSI} mutation in different subgroups of MSI CRC patients

Figure S9

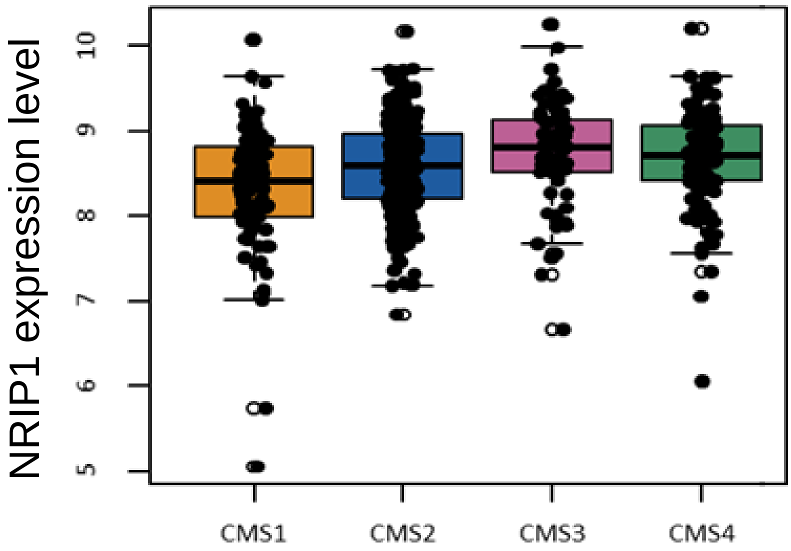

Subtype	Characteristics	Nb of samples	Tukey's p value vs CMS1
CMS1	MSI immune, BRAF mutations	91	n.a.
CMS2	Canonical WNT activation	232	0.0293
CMS3	Metabolic deregulation	69	0.0001
CMS4	Mesenchymal TGF-b activation	127	0.0002

***NRIP1* expression in the different CRC consensus molecular subtypes**

Figure S10

Table S1. Primer sequences

Gene Symbol	Forward Sequence	Reverse Sequence
**mLacZ**	CCGGTCGCTACCATTACCAG	CCGATTGTCTGTTGTGCC
**mNRIP1**	AGAACGCACATCAGGTGGCA	GATGGCCAGACACCCCTTTG
**mMsh2**	TGGCAGTTTTTGTGACTCCT	AAGGAACTCGTGGTTTTCCA
**mMsh6**	AGGCAAAGGATCTCAACGG	TTAGCCCAAACCAAATCACC
**hNRIP1**	AATGTGCACTTGAGCCATGATG	TCGGACACTGGTAAGGCAGG
**hMSH2**	TTCTGACTTCTCCAAGTTTCAGG	GGATCAAATGAAGGTTTTACAAGG
**hMSH6**	GCGAAGAACCTCAACGGAG	CAGGGGTAACCCTCCATCTT
**MSH2-7**	GCATGGGAGTAACATCAGAAGGA	CCGATGTTGGCAGTTTGCTT
**MSH6-2**	TTTCCGAGGCCTTTCGGGT	CTCCGACCCGGGGTTAGT
**MSH6-4**	AGCCCCGCGGCGTGAGG	GGCTGGCACACTGGTGGGTA
**hRIP^{MSI}**	GAGTACTTTCATCTCTTAAGGGAGTTT	CCAGTTGCTCCTGGGGAA
**siNRIP1**	GAAGCGUGCUAACGAUAAA	
**siMSH2**	UCCAGGCAUGCUUGUGUUGAA	
**siMSH6**	AUCGCCAUUGUUCGAGAUUUA	

Table S1

Table S2. Patients and tumor characteristics (IHC analysis)

Characteristic	n = 122<br>n (%)
Age at diagnosis<br>(median, years)	67<br>[21-89]
Sex	
Male	54 (51)
Female	52 (49)
Tumor site*	
Primary colon cancer	106 (87)
Metastasis	16 (13)
pTNM stage	
I	19 (16)
II	35 (29)
III	35 (29)
IV	14 (14)
MSI/MMR status	
MSS	12 (10)
MSI	12 (10)
MMR mutation	n.d.
No MMR mutation	n.d.
Unknown	98 (80)

* Multiple sites for 13 patients

Table S3. Summary of the effects of NRIP1 on the response to cytotoxic drugs in MEFs and HCT116 cells

Drug	IC_{50} ratios (MEFs) WT vs KO	p-value	IC_{50} ratios (HCT116) RIP140 vs GFP	p-value
Oxaliplatin [µM]	2.21	< 0.05	3.05	< 0.05
5-FU [µM]	2.03	< 0.01	2.65	< 0.001
SN38 [µM]	1.92	< 0.05	2.06	< 0.05

Table S4. Patient and tumor characteristics

Characteristic	Stage III CRCs n=63		Stage III dMLH1 CRCs n=41		Other CRCs n=153	
	Population n (%)	RIP^{MSI} n (%)	Population n (%)	RIP^{MSI} n (%)	Population n (%)	RIP^{MSI} n (%)
**NRIP1**						
Wild-type	54 (85.7)	/	36 (87.8)	/	115 (75.2)	/
Mutated	9 (14.3)	/	5 (12.2)	/	38 (24.8)	/
**Age**						
Age at diagnosis (median, years)	63 [21.8-97]		71 [29.3-97]		71.5 [20.7-93.9]	
<55	24 (38.1)	5 (20.8)	7 (17.1)	2 (28.6)	61 (39.9)	10 (16.4)
≥55	39 (61.9)	4 (10.3)	34 (82.9)	3 (8.8)	89 (58.2)	28 (31.5)*
Missing data	0 (0)	0 (0)	0 (0)	0 (0)	3 (2)	0 (0)
**Sex**						
Men	35 (55.6)	6 (17.1)	20 (48.8)	3 (15)	74 (48.4)	17 (23)
Women	28 (44.4)	3 (10.7)	21 (51.2)	2 (9.5)	79 (51.6)	21 (26.6)
**Tumor site**						
Colon	56 (88.9)	7 (12.5)	37 (90.2)	4 (10.8)	134 (87.6)	33 (24.6)
Rectum	7 (11.1)	2 (28.6)	4 (9.8)	1 (25)	17 (11.1)	5 (29.4)
Missing data	0 (0)	0 (0)	0 (0)	0 (0)	2 (1.3)	0 (0)
**pTNM stage**						
I	/	/	/	/	18 (11.8)	6 (33.3)
II	/	/	/	/	113 (73.9)	28 (24.8)
III	63 (100)	9 (14.3)	41 (100)	5 (12.2)	22 (14.4)	4 (18.2)
**BRAF V600E**						
No	29 (46)	3 (10.3)	14 (34.1)	2 (14.3)	77 (50.3)	15 (19.5)
Yes	16 (25.4)	3 (18.8)	16 (39)	3 (18.8)	31 (20.3)	11 (35.5)
Missing data	18 (28.6)	3 (16.7)	11 (26.8)	0 (0)	45 (29.4)	12 (26.7)
**MLH1 status**						
dMLH1	41 (65.1)	5 (12.2)	/	/	/	/
pMLH1	20 (31.7)	4 (20)	/	/	/	/
Missing data	2 (3.2)	0 (0)	/	/	/	/

*p-value = 0.01 in ≥55-year-old patients with stage III dMLH1 CRC versus other patients with CRC
